# Health Issues Among Nepalese Youth: A Literature Review

**DOI:** 10.7759/cureus.45108

**Published:** 2023-09-12

**Authors:** Veenah Stoll, Naomi Edwin, Kripa Dahal, John A Barnes, Miranda Pfautsch, Lahana Maharjan, Cyril Blavo

**Affiliations:** 1 Department of Medicine, Nova Southeastern University Dr. Kiran C. Patel College of Osteopathic Medicine, Clearwater, USA; 2 Department of Global Health, International Health Initiatives, Inc., Clearwater, USA; 3 Department of Public Health, Manmohan Memorial Institute of Health Sciences, Kathmandu, NPL; 4 Department of Public Health, Nova Southeastern University Dr. Kiran C. Patel College of Osteopathic Medicine, Clearwater, USA

**Keywords:** visual impairment, dental caries, malnutrition, mental health, water sanitation and hygiene, health issues, adolescent, child, nepal

## Abstract

Nepal is one of the world's least-developed countries. Nepalese children are often vulnerable to a lack of resources which leads to suboptimal levels of health in turn. This review article aims to identify health issues and inequities faced by school-aged children greater than five years old in Nepal. A comprehensive search of the literature was conducted in PubMed and Global Health databases to gather relevant studies. Inclusion and exclusion criteria were applied to select appropriate articles, and 35 full-length articles were reviewed in-depth. The literature supports the association between inadequate resource distribution among Nepalese children and poorer health outcomes compared to youth in developed countries. The key health issues of Nepalese youth identified in the literature consist of diarrheal illness, stunted growth, dental caries, visual impairment, poor mental health, and low health literacy. This review article aims to identify key health issues affecting Nepalese youth as well as propose interventions that can lead to an enhanced quality of life in this population.

## Introduction and background

Nepal is a beautiful mountainous nation in South-East Asia, bordering China and India. It is classified as a developing country, and health disparities remain common. Children, in particular, may suffer lifelong adverse effects due to poor access to resources during initial growth and development [1]. This paper seeks to assess the health issues, resources, and practices that impact Nepalese children and propose interventions that may enhance their health. Through education, health screening, strategic planning, and capacity-building aimed toward disease prevention, the quality of life of Nepalese children may be improved.

## Review

Methods

A search of the literature was conducted in the PubMed and Global Health databases. Search concepts included "Nepal," "health," and "youth." These search concepts were then modified for each database to employ database-specific search terms. Databases were searched in April 2021, and only articles published during or after 2016 were included. This search resulted in 294 articles. Our criteria included Nepalese school-aged children from five to 18 years old. Each title was then analyzed, and 225 titles that did not fit our criteria were excluded. Sixty-nine abstracts were then read to determine if the article met our criteria. Thirty-four full-length articles were then reviewed in-depth. No further articles were excluded during the full-text review. The paper was then organized into the following topics: diarrheal illness, stunted growth, dental caries, visual impairment, poor mental health, reproductive health challenges, and low health literacy.

Diarrheal illness

Randomized trials targeted low-middle income countries (LMICs), including Nepal, showed that 7% of deaths in school-aged children are associated with diarrheal diseases [1]. These deaths are attributed to unsafe water, inadequate sanitation, and poor hygiene (WASH) in 96% of cases [1]. Intestinal parasitosis is detected as a prevalent source of diarrheal infection in Nepal. Prevention should be the primary focus, with expected outcomes of good water quality and improved sanitation. Adequate water filtration and proper handling of meat, vegetables, and fruits are necessary to avoid intestinal infections [1]. A cross-sectional study conducted in Kathmandu Valley, Nepal, investigated the contamination of water, raw vegetables, and infected animal/meat products. Of the 196 children below the age of 15 years whose stool samples were tested for parasites, approximately 14% (13.7%) tested positive for parasitic infections [2]. An increased rate of diarrheal infection was also found in the summer and rainy seasons, with rates peaking in August [2].

*Taenia solium* is a parasitic tapeworm that is transmitted through ingestion of undercooked pork or poor hygiene [3]. Neurocysticercosis is a severe complication of this infection due to the hematogenous spread of the tapeworm larvae into the brain. A study was performed to identify Nepalese children with seizures due to neurocysticercosis. The investigators reviewed records of children zero to 17 years old from the Department of Pediatrics at the Manipal Teaching Hospital in Pokhara, Nepal [3]. Of the 1,355 patients with seizure disorders, 16.9% were due to neurocysticercosis, with a mean age of 9.7 years. Improved hygienic practices, including biannual deworming with albendazole, led to a decrease in cases of neurocysticercosis from 2003 to 2015. Risk factors of *Taenia solium* infection are similar to other diarrheal diseases and include the consumption of undercooked meat, improper or absent food inspection, poor sanitation, and use of untreated human waste as crop fertilizer [3].

WASH problems were the cause of 19% of child mortality worldwide, and 3,500 child deaths were a result of waterborne disease each year in Nepal [4]. A water quality study in Nepal was performed using a sample size of 800 children from 16 schools and student households. Ten percent of the water sources tested were from rural communities. The study revealed that domestic animals were kept in over 90% of households, and 86% of households did not use treated water sources. Schools reported not using treated water sources, and 12% of students did not practice basic hygiene, such as washing their hands before eating or after using the restroom [4]. Results from another survey in the districts of Dolakha and Ramechhap showed that more than 50% of families had inadequate disposal of wastes, 77% of water samples were contaminated, and the prevalence of intestinal parasitic infections was approximately 40% [5].

Teachable hygiene practices, better sanitation while preparing food, and water decontamination techniques are essential to decreasing the prevalence of parasitic infections in children. An intervention in two Nepalese districts that implemented a school garden, WASH practices, education, health, and nutrition interventions for students between the ages of eight and 17 years was done with a 15-month follow-up. The WASH intervention group showed a decreased prevalence of stunting and anemia with improved hygiene practices [6]. Another study performed in 2013 implemented school health and nutrition programs by trained teachers. These programs included regular health check-ups for students, iron and vitamin A supplementation, mass deworming, provision of a mid-day meal, separate and adequate toilets, access to first aid kits, and promotion of a tin-box library or information, education, and communication corner [7]. The control group only had access to traditional government-sponsored resources, including basic health education curricula, hygiene and sanitation facilities, and deworming programs. After a year of implementation, the intervention group demonstrated a significantly lower prevalence of infectious diarrhea and dysentery [7].

Stunted growth

Nutrition is a vital aspect of development in children around the world. Malnutrition can impair growth and development, increase disease through a weakened immune system or higher pathogenic burden, and cause childhood deaths in LMICs. The Nepal Demographic and Health Survey conducted from 1996 to 2016 identified stunted growth as a major outcome of undernutrition [8]. Overall, 36% of children under the age of five years were stunted, 12% were severely stunted, 10% were wasted, 2% were severely wasted, 27% were underweight, and 5% were severely underweight. Only 1% of the children were overweight [8]. Stunted growth is also associated with poor maternal nutrition leading to low birth weight in their newborns. This is prevalent in the poorer wealth quintiles of Nepal where there are high rates of poverty, low maternal education, and poor dietary practices [8].

WASH interventions, combined with school gardens, have had significant effects on child health parameters. A study performed in 2015-2016 used 12 schools that were randomly divided into three groups: a school garden program, a school garden program with complimentary WASH, and a control group. The study found that schools that implemented a school garden program and a WASH program demonstrated improved nutritional knowledge. Knowledge regarding the importance of eating fruits and vegetables led to an increase in household consumption of these foods. There was also a significant reduction in parasitic infections compared to controls and a slight decrease in growth stunting [6]. Furthermore, another cross-sectional study, conducted on 440 students in selected primary schools in Kathmandu, analyzed the association between access to green spaces and obesity prevalence. This study found that the distance to green space was the most significant contributing factor to decreasing the development of childhood obesity, showing its importance [9].

Stunted growth among Nepalese children has declined between 2011 and 2016, even in the poorest households. The decline in growth stunting was the highest in households where the mother received some primary education [10]. A correlation between maternal thinness, anemia, and low birth weight with anemia, stunting, and wasting was established in the Torlesse and Aguayo overview paper commissioned by UNICEF in 2016-2017 [11]. The occurrence of anemia among adolescent females aged 15 to 19 rose from 38.5% in 2011 to 43.6% in 2016, according to the Nepal Department of Health Services [12]. Young adolescent girls (10-14.9 years old) had the highest prevalence of being underweight (45%), while adolescent mothers (15-19.9 years old) had the lowest prevalence (19%). Anemia was statistically higher in these adolescent mothers as well with 29% of these individuals being affected [13]. These results suggest there may be inadequate knowledge of fundamental nutrition concepts, knowledge application issues, or that both may be factors. Anemia in children adversely affects their physical growth and may prevent them from reaching their full potential for educational achievement and labor productivity [14]. Early identification of malnutrition in school children allows stakeholders to promote better health practices, reduce the impact of infections, and provide children with life skills that could enhance their long-term health. Interventions are more effective when implemented at multiple levels, including individual, school-wide, and community-wide [1]. Understanding the driving forces that lead to undernutrition is key to effective policy, advocacy, and programming. This could likely be achieved through strengthening the multi-sector approach to nutrition, WASH interventions, agriculture, and education access [6].

Dental caries

Dental hygiene is an important indicator of health. A descriptive cross-sectional study was performed in 2019 in a private school located in the Panauti municipality of Nepal to investigate common morbidities of students in grades one through 10 (n=356). The results showed that dental caries were the most prevalent [15]. Another study was done to determine if there was an association between untreated dental caries and BMI. This cross-sectional study was performed in 18 out of 75 districts in Nepal. Age groups recruited for the study included five- to six-year-olds, 12-year-olds, and 15-year-olds with a total sample size of n=1,135 [16]. A questionnaire was administered to the children to assess both oral hygiene and diet. Poor diet was associated with poor dental hygiene and increased dental caries, while improved nutrition and good oral hygiene enhanced dental health. Results showed both underweight and overweight BMI were related to poor diet [16]. Karki et al. analyzed samples from children with untreated dental caries using four categories based on the severity of the caries. A positive association was identified in the Newari ethnic group between a higher quantity of dental caries and patients with lower oral health-related quality of life, lower performance in school, and higher absenteeism [17].

Visual impairment

Preventable vision loss can create many disadvantages for those affected. A pediatric ophthalmologist evaluated 778 Nepalese students in integrated schools to determine common causes of blindness or visual impairment. It was found that 85.9% of the children were blind, 10% had severe visual impairment, and 4.1% were visually impaired [18]. Of those who were blind, 40.9% of students had potentially avoidable causes of blindness with 23.1% being preventable and 17.8% being treatable. Some of the preventable causes found in this study included corneal blindness due to vitamin A deficiency, measles, and harmful traditional eye practices. The treatable causes included cataracts and glaucoma [18]. This study demonstrates the need for better eye health awareness. The VISION 2020 initiative in Nepal reported a reduction in the prevalence of blindness from 0.84% to 0.35%, which was attributed in part to the decline in blinding xerophthalmia and trachoma [12]. Proposed interventions include advocacy for eye care and early detection through regular eye screenings for children [18].

Poor mental health

Mental health is a critical factor of child development that should be prioritized while young brains are still developing. The brain is highly malleable during childhood and early adolescence making any encounter, positive or negative, during these formative years important in how they view the world. Aryal et al. described a lingering stigma toward mental health that is still held by the older generations in Nepal. Some communities hold a belief that mental health challenges are equivalent to "insanity" or associated with “sins perpetrated in the past life” [19]. This stigma prevents adolescents from seeking treatment, further isolating these individuals [19]. The threats of natural disasters [20], food insecurity [21], and child marriages [22] are not uncommon in Nepal. These factors may also play a role in the increased incidence of poor mental health.

In a 2019 health survey sampling 6,531 Nepalese students in 74 schools, more than half of the children surveyed experienced bullying for at least one day in the last 30 days (50.7%). Approximately 14% reported suicidal ideation or plan and 11% attempted suicide [20]. Children who were bullied were at five times increased risk of substance abuse and 11 times increased risk of physical violence [23].

Post-traumatic stress disorder (PTSD) can be triggered by a variety of traumatic stimuli. In Nepal, post-earthquake aftermath and being conscripted as a child soldier are known triggers of PTSD. Two post-earthquake PTSD studies were done in the Kathmandu district where researchers reported PTSD rates of 10.7% and 51% [24]. Child soldiers less than 18 years of age were also found to be more likely to exhibit PTSD symptoms at a rate of 55.3% compared to 20% in non-child soldiers [24]. Former child soldiers suffer not only from PTSD at a higher incidence but also from depression and anxiety. One study reported symptoms of depression in 53.2% of child soldiers as compared to 24.1% of non-child soldiers. Anxiety symptoms were present in 46.1% of child soldiers as compared to 37.6% of non-child soldiers [24].

Female children in Nepal are more susceptible to additional stressors that include sexual harassment, childhood marriage, and stigma regarding menses [22,25-27]. The use of public transportation is strongly correlated with sexual harassment in Kathmandu Valley, particularly during evening hours. Females who are sexually harassed often reflect on negative thoughts and develop low self-esteem, loneliness, hopelessness, and depression, which may lead to self-harm or suicide [25]. Implementing educational programs that address sexual harassment can be vital in reducing the adverse impact on young females.

Childhood marriage also contributes to the mental health burden of adolescents in Nepal. Nepal has one of the highest prevalence of child marriage prior to the age of 18 years [22]. Marriage is viewed as an essential stage in life with strong social sanctions against childbearing unless married. However, underage marriages can lead to physical and emotional strain for adolescent girls. Early marriage and sexual initiation have also been linked to potential complications during childbirth at an early age. For example, there is an increased risk of fistula development in adolescent mothers which can be a debilitating condition and lead to social exclusion. Adolescent mothers are less likely to be educated, wealthy, or in urban dwellings and are at an increased risk of inadequate antenatal care [22]. The Nepalese government signed a charter in 2014 with the goal to end all child marriages by the year 2020. They launched a “National Strategy to End Child Marriage (Human Rights Watch 2016)” to empower, educate, and provide services to girls. This has led to a decrease in childhood marriage; however, there is no evidence to support the complete eradication of this practice. Despite the barriers these girls face, there is an opportunity to build on recent achievements that promote child well-being and gender equality through reductions in child marriage [26].

Reproductive health challenges

Reproductive health is another important factor that should be considered when assessing the overall health of female adolescents. Menstrual hygiene practices, perceptions of these practices, feelings of shame and distress, and management of bleeding and odor have been found to influence the mental health of adolescent girls [27]. The limited knowledge and social support regarding menstruation are often constrained by widespread stigmas and gender norms that consider discussions on such topics inappropriate. These stigmas and norms also contribute to shaping behavioral expectations, which are both externally imposed and internalized by individuals. Additionally, the lack of access to affordable menstrual materials emerges as a significant barrier to attaining adequate health and hygiene for many girls. The cumulative effect of these experiences has been shown to harm their physical and psychological health as well as their education and social engagement [28]. Restrictions, like those imposed on daily tasks, create an even greater mental and psychological strain during menstruation. In a study with 1,342 adolescent female participants, more than half of women viewed menstruation as a “bother” or “curse” [28] The Hindu women who were surveyed reported abstaining from praying and other religious activities. However, members of the Janajati caste or women who held a master’s degree were more likely to enter places of worship and pray during their menses [29]. Although 89% of women reported restrictions for food preparation, religious activity, and social interactions while menstruating, 60% of females reported positive menstrual health attitudes and practices [29]. These seemingly discordant findings showcase that similar environmental factors can have different individual outcomes and should not be used as the sole indicator in assessing the overall reproductive and mental health of adolescents [29]. The inequalities in female reproductive health within Nepal underscore the enduring influence of deeply rooted cultural and religious superstitions in everyday life.

A comparison of menstrual knowledge was made among the six most prevalent castes/ethnic groups in Nepal. While 91% of respondents considered themselves to have good menstrual health knowledge, less than half were able to correctly answer more than two out of four questions regarding basic menstrual health. The Brahman/Chhetri class and those living in the Tarai (lowlands) districts were more likely to have high menstrual health knowledge when compared to the Janajati caste or those living in the hill districts [29]. A similar study to assess menstrual hygiene knowledge was performed in the Dang district of Nepal, and this study considered good knowledge to be a grade of ≥7 out of 12 questions. This questionnaire showed that 87.7% of adolescent girls had good knowledge of menstrual health, but only 67% were able to apply these practices. The questions with the least correct responses involved the etiology and age of menopause [30]. Factors such as maternal literacy, father’s education to grade 10, private school attendance, smaller family size ≤four, and living with relatives were significantly associated with increased menstrual health awareness [30,31]. An association was found between poorer menstrual health awareness and rural living or living alone with their mother [31].

Health facilities caring for adolescents require resources to address the complex sexual and psychosocial needs of adolescents. The successful use and effectiveness of 26 adolescent-friendly health services (AFHS) were assessed using a qualitative approach. Despite these AFHS providing an ideal avenue for adolescents to access reproductive healthcare, Nepal’s socio-cultural stigmas make creating a truly adolescent-friendly environment difficult. The assessment revealed healthcare providers were frequently allowing their personal beliefs and judgments to influence the healthcare they provided. This limited the application of ethical and patient-centered care styles necessary to fully cater to the needs of these adolescents [32]. Cunningham et al. surveyed their use and effectiveness, and they found these clinics were not very popular, with only 5% of girls having ever visited one. Efforts aimed at addressing stigmas can contribute to improving the healthcare environment within clinics [13].

Similarly, these stigmas regarding sex have an implication for sexual health knowledge. These implications were demonstrated in the results of a sexual health knowledge assessment performed in secondary school-aged children. It concluded adolescents were interested in four main themes: (1) curiosity and desire to know about sex, (2) the communication gap with parents, teachers, and seniors, (3) the influence of local customs, media, and peer pressure, and (4) sexual and reproductive health knowledge and services [33]. An inclusive and informal environment was found to be the most comfortable for participants when broaching discussions on this subject. It was also notable that females included in the study indicated that they were often uncomfortable when speaking with teachers about sexual and reproductive health [33]. These results suggest that educational programs may be helpful to fill this gap.

To assess the effectiveness of current school-based sex education programs, a study was performed in secondary schools in urban Kathmandu Valley. The most heavily discussed topic was susceptibility to contracting HIV/AIDS and sexually transmitted infections (STIs). However, students reported knowing the least about where HIV counseling and testing can be received and communicating with parents or other trusted adults about sexual topics. The topic evaluating parent and teacher support identified a lack of participation in sexual health education by parents/guardians [33].

To ensure the most effective understanding of the risk of contracting STIs or HIV/AIDS, it is important to provide comprehensive information about testing and counseling concurrently. The effectiveness of such interventions has already been tested and proven. When given adequate education regarding HIV and reproductive health, there was a significant association between positive attitudes regarding abstinence and safer sex [34]. While school-based sex education programs are not always an option, other solutions like peer education, education of young girls by their mothers, and further training of school staff can improve sexual awareness and decrease related anxiety and depression. These findings portray a significant need for improved communication between parents, schools, and adolescents to foster an open and safe environment and establish self-esteem and trust.

Low health Literacy

Health literacy disparities in LMICs can present in numerous ways. The health literacy gaps identified among the youth in the Kathmandu Valley of Nepal encompass areas such as common health problems, nutrition, and menstruation [8,27] as discussed throughout the paper. Acquiring fundamental knowledge in these domains is crucial for personal empowerment, particularly concerning menstrual health awareness among adolescent girls. It is important to assess the understanding of these general conditions at a population level so that personalized educational programming and government policy can be developed. After a thorough analysis of the current gaps in understanding, updated guidelines can be established with the greatest potential to improve the quality of life for Nepalese youth.

Proposed interventions

Implementing sustainable interventions will ideally lead to better health outcomes for children who then become healthy adults with the capacity to raise healthy children. Deworming programs, proper sanitation in schools and communities, and education on hygiene practices can significantly reduce the burden of diarrheal illnesses [3]. Additionally, the high prevalence of stunted growth and anemia implies a need for comprehensive interventions that focus on nutrition education, access to nutritious food, and maternal health. Implementing school garden programs, WASH interventions, and nutrition education can improve nutritional knowledge and practices among children and promote healthy growth and development [1].

Dental caries and visual impairment are also health concerns faced by Nepalese children. Poor oral hygiene and diet contribute to dental caries, emphasizing the importance of promoting good oral hygiene practices and healthy dietary habits [14,15]. Visual impairment, often preventable, calls for increased access to vision screenings and corrective eyeglasses in early childhood [17].

Addressing poor mental health and low health literacy among Nepalese youth is vital. Mental health support services and open discussions can help reduce this burden among Nepalese children [19]. Nepal currently lacks sufficient child and adolescent mental health services and policies and would benefit from innovative interventions [22]. Sustainable mental health services that are sensitive to adolescents' experiences of trauma and their unique needs would be a necessary component of long-term rehabilitation [19].

Reproductive health among Nepalese youth also has its challenges. It is crucial to tackle stigmas, enhance knowledge, and foster supportive environments. This can be accomplished through inclusive cultural practices, targeted education programs, and improved healthcare settings [12]. Comprehensive sex education, better communication among parents, schools, and adolescents, and initiatives to prevent HIV/AIDS and STIs are vital for promoting the overall well-being of Nepalese youth [33]. Improved maternal literacy could be another avenue to enhance good menstrual health practices in their daughters [30]. Educational programming that targets men and boys could also help reduce these stigmas [28].

## Conclusions

The youth of Nepal are susceptible to many health issues, including diarrheal illness, stunted growth, dental caries, visual impairment, poor mental health, and low health literacy. These health issues are often associated with inadequate resource distribution, inefficient sanitation practices, and lack of access to healthcare and education. Educational programs and awareness campaigns could enhance health literacy and empower adolescents with the knowledge to make informed decisions about their health. Investigating the root causes of health-related problems children face is key to finding solutions that create lasting change in Nepalese communities. Further areas that require investigation include physical health, determination of children's average activity levels, and sleep hygiene. This would provide further insight into the inequities children of Nepal experience, enabling stakeholders to create well-rounded programs designed to improve child health. These early interventions focused on preventative measures could profoundly impact the lives of Nepalese children.
